# Supplementary figures and images for: Sequence variant analysis of RNA sequences in severe equine asthma
Source: PeerJ. 2018 Oct 11;6:e5759. doi: 10.7717/peerj.5759 (PMC6186407; doi:10.7717/peerj.5759)

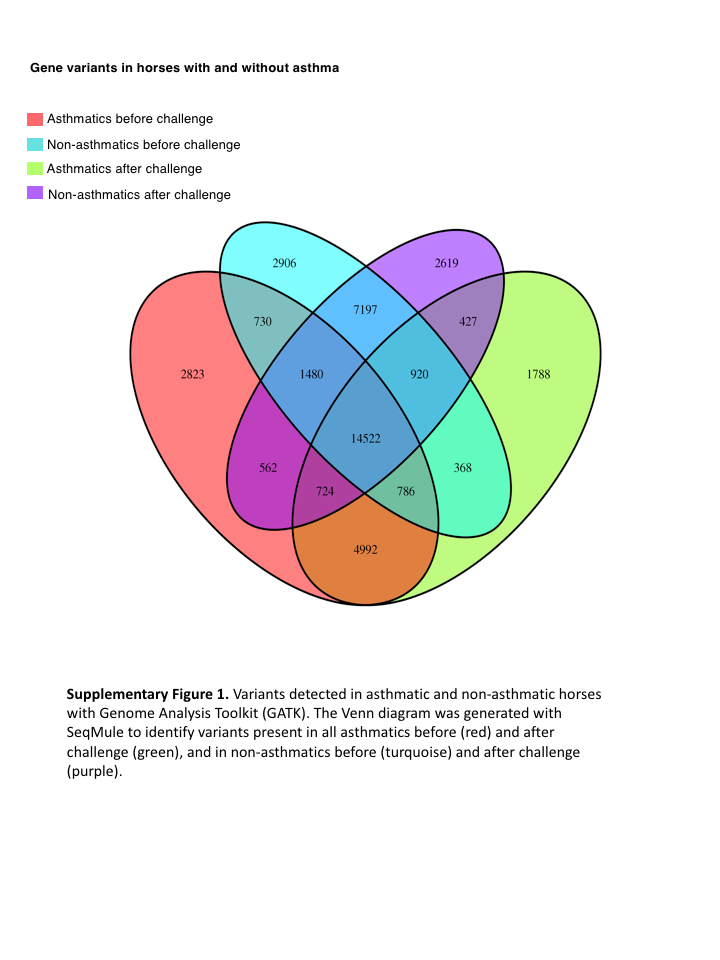

Supplement: Supplemental Information 1 — The Venn diagram was generated with SeqMule to identify variants present in all asthmatics before (red) and after challenge (green), and in non-asthmatics before (turquoise) and after challenge (purple). [file peerj-06-5759-s001.png]

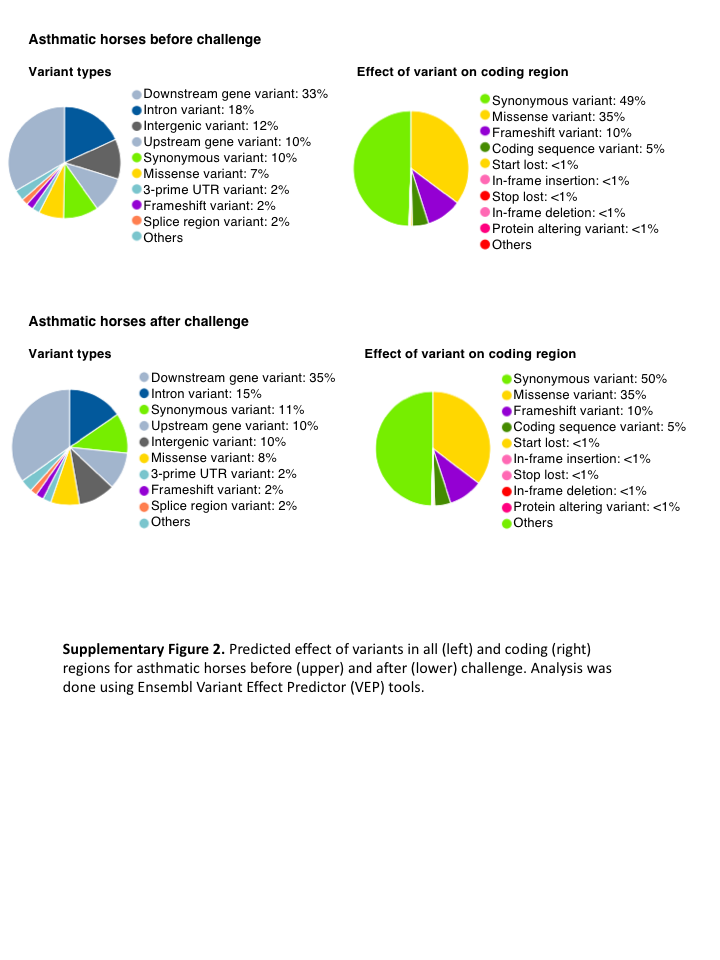

Supplement: Supplemental Information 2 — Analysis was done using Ensembl Variant Effect Predictor (VEP) tools. [file peerj-06-5759-s002.png]

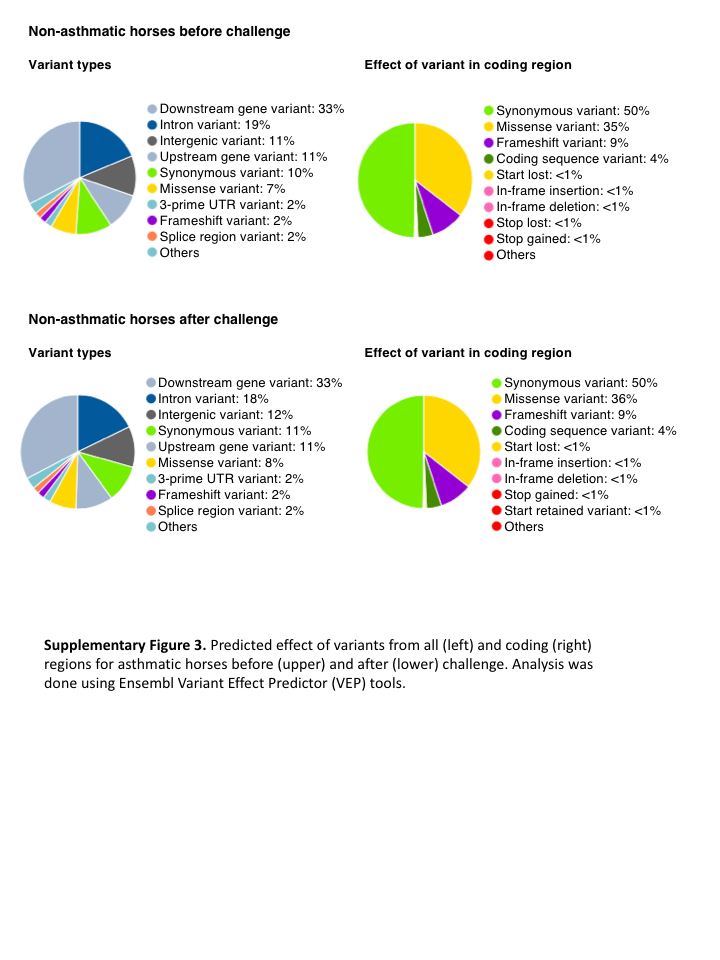

Supplement: Supplemental Information 3 — Analysis was done using Ensembl Variant Effect Predictor (VEP) tools. [file peerj-06-5759-s003.png]

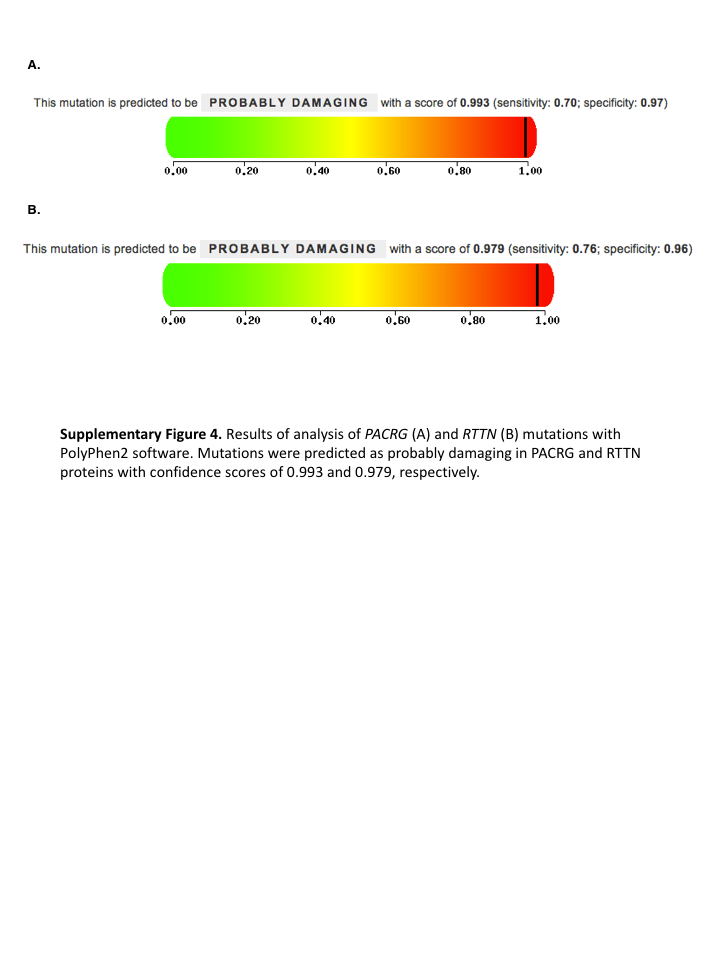

Supplement: Supplemental Information 4 — Mutations were predicted as probably damaging in PACRG and RTTN proteins with confidence scores of 0.993 and 0.979, respectively. [file peerj-06-5759-s004.png]
