# Supplementary material for: Sequence variant analysis of RNA sequences in severe equine asthma
Source: PeerJ. 2018 Oct 11;6:e5759. doi: 10.7717/peerj.5759 (PMC6186407; doi:10.7717/peerj.5759)
Supplement: Supplemental Information 5 [file peerj-06-5759-s005.docx]

Table 1. Predicted effect of amino acid substitution at position 182 in PACRG

| **Variant** | **Outcome** | **Score** | **Accuracy** |
| --- | --- | --- | --- |
| V182A | neutral | -1 | 53% |
| V182R | effect | 85 | 91% |
| V182N | effect | 75 | 85% |
| V182D | effect | 81 | 91% |
| V182C | effect | 34 | 66% |
| V182Q | effect | 78 | 85% |
| V182E | effect | 75 | 85% |
| V182G | effect | 78 | 85% |
| V182H | effect | 82 | 91% |
| V182I | neutral | -59 | 78% |
| V182L | effect | 65 | 80% |
| V182K | effect | 86 | 91% |
| V182M | effect | 64 | 80% |
| V182F | effect | 66 | 80% |
| V182P | effect | 83 | 91% |
| V182S | effect | 32 | 66% |
| V182T | effect | 51 | 75% |
| V182W | effect | 84 | 91% |
| V182Y | effect | 79 | 85% |
| V182V | neutral | -85 | 93% |
