# Supplementary material for: Sequence variant analysis of RNA sequences in severe equine asthma
Source: PeerJ. 2018 Oct 11;6:e5759. doi: 10.7717/peerj.5759 (PMC6186407; doi:10.7717/peerj.5759)
Supplement: Supplemental Information 6 [file peerj-06-5759-s006.docx]

Table 2. Predicted effect of amino acid substitution at position 1807 for RTTN

| **Variant** | **Outcome** | **Score** | **Accuracy** |
| --- | --- | --- | --- |
| R1807A | effect | 44 | 71% |
| R1807R | neutral | -99 | 97% |
| R1807N | effect | 54 | 75% |
| R1807D | effect | 80 | 91% |
| R1807C | effect | 64 | 80% |
| R1807Q | effect | 34 | 66% |
| R1807E | effect | 67 | 80% |
| R1807G | effect | 55 | 75% |
| R1807H | effect | 43 | 71% |
| R1807I | effect | 62 | 80% |
| R1807L | effect | 49 | 71% |
| R1807K | effect | 21 | 63% |
| R1807M | effect | 60 | 80% |
| R1807F | effect | 74 | 85% |
| R1807P | effect | 84 | 91% |
| R1807S | effect | 45 | 71% |
| R1807T | effect | 50 | 75% |
| R1807W | effect | 81 | 91% |
| R1807Y | effect | 67 | 80% |
| R1807V | effect | 66 | 80% |
