# Supplementary material for: Sequence variant analysis of RNA sequences in severe equine asthma
Source: PeerJ. 2018 Oct 11;6:e5759. doi: 10.7717/peerj.5759 (PMC6186407; doi:10.7717/peerj.5759)
Supplement: Supplemental Information 7 [file peerj-06-5759-s007.docx]

Table 3. Allelic frequencies for *PACRG* and *RTTN* in asthmatic and non-asthmatic horses

|  | **Allele frequency** | |
| --- | --- | --- |
| ***PACRG*** | Asthmatics | Non-asthmatics |
| A | 12 | 16 |
| G | 8 | 12 |
| χ^2^ | 9.20^e-31^ | |
| *P*-value | 1 | |
| ***RTTN*** |  |  |
| A | 9 | 4 |
| T | 11 | 24 |
| χ^2^ | 4.1263 | |
| *P*-value | 0.042 | |
