# Supplementary material for: Sequence variant analysis of RNA sequences in severe equine asthma
Source: PeerJ. 2018 Oct 11;6:e5759. doi: 10.7717/peerj.5759 (PMC6186407; doi:10.7717/peerj.5759)
Supplement: Supplemental Information 8 [file peerj-06-5759-s008.docx]

Table 4. Pearson’s residual for *PACRG* and *RTTN* allele frequency calculation in asthmatic and non-asthmatic horses

|  | **Pearson’s residual** | |
| --- | --- | --- |
| ***PACRG*** | Asthmatics | Non-asthmatics |
| A | 0.098 | -0.082 |
| G | -0.115 | 0.098 |
| ***RTTN*** |  |  |
| A | 1.540 | -1.301 |
| T | -0.938 | 0.793 |
