# Supplementary material for: Sequence variant analysis of RNA sequences in severe equine asthma
Source: PeerJ. 2018 Oct 11;6:e5759. doi: 10.7717/peerj.5759 (PMC6186407; doi:10.7717/peerj.5759)
Supplement: Supplemental Information 9 [file peerj-06-5759-s009.docx]

Table 5. Genotype frequency for *PACRG* and *RTTN* in asthmatic and non-asthmatic horses

|  | **Asthmatics** | **Non-asthmatics** |
| --- | --- | --- |
| ***PACRG* genotype** | Observed | Observed |
| A/A | 2 | 5 |
| A/G | 8 | 6 |
| G/G | 0 | 3 |
| *P*-value | 0.213 | |
| ***RTTN* genotype** | | |
| A/A | 2 | 0 |
| A/T | 5 | 4 |
| T/T | 3 | 10 |
| *P*-value | 0.055 | |
