# Supplementary material for: Sequence variant analysis of RNA sequences in severe equine asthma
Source: PeerJ. 2018 Oct 11;6:e5759. doi: 10.7717/peerj.5759 (PMC6186407; doi:10.7717/peerj.5759)
Supplement: Supplemental Information 10 [file peerj-06-5759-s010.docx]

**Sequence alignment and protein secondary structure prediction**

DNA sequence alignment and prediction of secondary structure was performed with Geneious version 10.2.3. Default settings were used for both operations. Hydrophobicity and isoelectric point (pI) were included for each amino acid of the protein sequence.

**Allele and genotype frequency**

Statistical differences in allele frequency between asthmatic and non-asthmatic horses for *PACRG* and *RTTN* were tested in R (version 3.3.3) using a Pearson's Chi-squared test with Yates' continuity correction with a 2x2 contingency table. Difference in genotype frequency was calculated using the Fisher's exact test for count data with a 2x3 contingency table. *P-*values <0.05 were considered significant.

**Qualitative prediction of variants of interest**

Sequence variants of interest were first identified based on presence in pre- and/or post-challenge samples from asthmatic horses. The STATS function in the SeqMule software [43] was used to identify consensus sequence variants within groups with the *–c -vcf* options. Venn diagrams depicting sequence variants shared between and within groups were constructed using the *-p –venn* options (Suppl. Figure 1). The Ensembl Variant Predictor (EVP) on-line tool [44] was used to further analyze and filter sequence variants. In asthmatic horses, a total of 26,619 pre- and 24,527 post-challenge sequence variants were identified, respectively, while the corresponding numbers were 28,909 and 28,451 for non-asthmatic horses. Difference in sequence variants before and after challenge could stem from differences in allele expression, alternative splicing or global gene expression. We previously reported a difference in gene expression between asthmatic and non-asthmatic horses following challenge, which may contribute to the observed differences [39]. Approximately 30% of sequence variants were novel and not previously described. The types of sequence variants and their coding region effects are summarized in Suppl. Figures 2 and 3. For further selection using VEP the inclusion criteria were 1) missense sequence variants in protein-coding sequence; and 2) predicted to cause loss of protein function. The effect of the sequence variant on protein function was analyzed with the Sorting Intolerant From Tolerant (SIFT) [45-49] tool with the threshold score set at <0.01. Low confidence loss of function predictions and existing sequence variants were excluded. Ten variants were present in all asthmatic horses (before or/and after challenge) but not all non-asthmatic horses. After manual verification, only two sequence variants were expressed in all asthmatic samples, but in only some non-asthmatic samples. Effects of these two substitution variants were then investigated with Polymorphism Phenotyping (PolyPhen) 2 [50] and Screening for Non-Acceptable Polymorphism (SNAP) 2 softwares [51-53].

**Results**

**Predicted effect of substitution variants on protein function**

The substitution variants detected in PACRG and RTTN were predicted to cause loss of protein function when analyzed with SIFT and PolyPhen2 (Suppl. Fig. 3A and B). For PACRG, the substitution variant score was 0.993 with sensitivity of 0.70 and specificity of 0.97. For RTTN, the score was 0.979 with a sensitivity of 0.76 and a specificity of 0.96. Furthermore, for PACRG, V to M substitution at position 182 was predicted to affect protein function with a score of 64 at 80% expected accuracy (Suppl. Table 1). For RTTN, R to W substitution at position 1807 would affect protein function with a score of 81 at 91% expected accuracy (Suppl. Table 2).

**Allele and genotype frequency for *PACRG* and *RTTN***

For *PACRG*, the frequency of the WT (G) and altered (A) alleles were not significantly different between asthmatic and non-asthmatic (χ^2^ =9.20^e-31^, *p* =1) animals, but for RTTN the WT (T) and altered (A) alleles were significantly different with χ^2^ =4.1263 and *p* =0.042 (Suppl. Table 3). The Pearson’s residuals confirmed that the altered allele (A) contributed most of the difference between the groups for *RTTN*, and had the strongest positive association with the asthmatic group, and the strongest negative association with non-asthmatics (Suppl. Table 4). Differences in PACRG and RTTN genotype frequency between groups were not significant (Suppl. Table 5).
